# Non-enzymatic formation of isoprene and 2-methyl-3-buten-2-ol (2-MBO) by manganese

**DOI:** 10.1038/s41598-022-06520-0

**Published:** 2022-02-14

**Authors:** Hirosuke Oku, Ishmael Mutanda, Masakazu Fukuta, Masashi Inafuku

**Affiliations:** 1 Tropical Biosphere Research Center, University of the Ryukyus, Okinawa, Japan; 2 The United Graduate School of Agricultural Sciences, Kagoshima University, Kagoshima, Japan; 3 Biofuels Institute, School of the Environment and Safety Engineering, Jiangsu University, Zhenjiang, Jiangsu China; 4 Faculty of Agriculture, University of the Ryukyus, Okinawa, Japan

**Keywords:** Biochemistry, Plant sciences, Environmental sciences

## Abstract

It has been suggested that isoprene synthesis by isoprene synthase (IspS) proceeds via a substrate-assisted mechanism. The authors observed a non-enzymatic isoprene formation by Mn^2+^, which represents the basis of IspS enzyme reaction. Because IspS and many other terpene synthases require Mn^2+^ metal ions as cofactor, this study characterized the formation reaction for the first time. Metal ions including Mn^2+^ non-enzymatically produced both isoprene and 2-methyl-3-buten-2-ol (2-MBO) from dimethylallyl pyrophosphate (DMADP). Isoprene formation was most enhanced by Fe^2+^ and, to a lesser extent, by Mn^2+^ or Cu^2+^. Ni^2+^, Co^2+^, Mg^2+^, and Ba^2+^ exhibited a low activity to generate both isoprene and 2-MBO. The proportion of isoprene and 2-MBO varied with the Mn^2+^ concentration: isoprene predominated over 2-MBO at a higher Mn^2+^ concentration. Similarly, isoprene formation by Mn^2+^ increased exponentially as temperature increased with predominance of isoprene over 2-MBO at higher temperature. Both isoprene and 2-MBO formation was enhanced by acidic and neutral pH compared to alkaline conditions. Molecular dynamic simulation of DMADP suggested that the formation reaction is initiated by deprotonation of hydrogen on allyl terminal carbon by phosphate oxygen and generates carbocation and allyl anion intermediates. This is followed by quenching to produce isoprene or by hydroxyl addition to form 2-MBO. Thus, this study provided an insight into reaction mechanism of isoprene and 2-MBO biosynthesis and highlighted some parts of isoprene emission from terrestrial plants, which could be formed by non-enzymatic mechanism.

## Introduction

Isoprene (2-methyl-1,3-butadiene) emitted from a terrestrial plants is an essential and the most abundant component in tropospheric chemistry^1–3^. Isoprene has a great impact on the chemistry of atmosphere through reaction with hydroxy radicals and nitrogen oxide leading to production of ozone or to increased life span of the greenhouse gas methane, while isoprene is considered to confer some advantages to plants including better stabilization of photosynthetic apparatus under stressed conditions^4^.

In addition, it is a precursor in the synthetic chemistry industry for the production of rubber, pharmaceuticals, and potential biofuel^5–7^. Isoprene for industrial chemistry has been mainly obtained from petrochemical sources and its supply is dependent on the petroleum industry wherein the production is energy consuming and environment-unfriendly. Bio-generation of isoprene using isoprene synthase (IspS) therefore has been attracting attention to overcome above mentioned disadvantages in chemical process^7,8^.

Furthermore, volatile organic compounds in breath can be a biochemical probe providing both non-invasive and continuous information on the metabolic and physiological state of an individuals. In terms of human exhalation, isoprene accounts for up to 70% of total hydrocarbon removal via exhalation and may act as a noninvasive indicator of several metabolic effects in the human body^9^.

In plants and bacteria, isoprene is biosynthesized by isoprene synthase^10–12^ whilst its formation mechanism in the human body remains unclear^9^. IspS and many other terpene synthases require Mg^2+^ or Mn^2+^ metal ions as a cofactor^13–17^. The enzyme rection of terpenoid biosynthesis depends for activation on a trinuclear cluster, usually containing Mg^2+^ or Mn^2+^. This cluster not only activate the reaction, but also control product specificity of the enzymes^18^. The authors have studied the metal requirements of IspSs in a series of gene cloning and characterization of IspS from tropical trees and found that these IspSs were most activated by Mn^2+^ or Mg^2+^ similarly to other terpenoid synthases^19^. During the study, the authors observed non-enzymatic formation of isoprene by divalent cations, especially to a greater extent by manganese ions (Mn^2+^). It has been implicated that isoprene synthesis by IspS proceeds via a substrate-assisted mechanism mediated by diphosphate oxygen as a base^20^. Thus, the substrate-assisted catalysis of isoprene formation constitutes the basis of the IspS enzyme reaction. The authors of this study characterized the non-enzymatic formation of isoprene by Mn^2+^ and investigated the reaction mechanism via molecular dynamic simulation for the first time.

## Materials and method

### Reagents

Dimethylallyl pyrophosphate (DMADP) was purchased from Merk Japan (Tokyo, Japan). The metal salts used include MnCl_2_∙4H_2_O, MgCl_2_∙6H_2_O, FeSO_4_∙7H_2_O, CuCl_2_∙2H_2_O, NiCl_2_∙6H_2_O, CoCl_2_∙6H_2_O, BaCl_2_∙2H_2_O, CaCl_2_, KCl, and NaCl. Guaranteed reagents were used, which were purchased from Nacalai Tesque (Tokyo, Japan) or Fujifilm Wako Pure Chemical Corporation (Tokyo, Japan).

### Formation of isoprene

The reaction mixture consisted of 45-mM Tris–HCl buffer (pH 8.5) containing 5% glycerol, 2-mM DTT, 10-mM metal ions, and 10-mM DMADP in 100 μL, unless otherwise specified. Isoprene formation was measured in 2-mL glass vials with a silicon septum cap. DMADP and metal ions were mixed on ice, and the formation reactions were induced by incubating the reaction mixture at 40 °C and then stopped by removing the reaction mixture from the vials on ice. The headspace gas of the reaction mixture (100 µL) was analyzed using a gas chromatograph (Agilent GC6890N or Shimadzu GC 14A) equipped with a flame ionization detector. The samples spit by the ratio of 5:1 were analyzed on DB-VRX columns (0.25 mm × 30 m, Agilent Technologies, CA, USA) at an isocratic constant temperature of 60 °C with helium as the carrier gas and flow rate of 30 cm/s. The chemical structure of the reaction product was identified by comparing the retention time with authentic standard or via gas chromatography–mass spectrometry (GC–MS) analysis using Shimadzu QP-2010. The columns and separation conditions of the GC–MS analysis were the same as those of gas chromatography. Ionization for GC–MS was by electron impact at 70 eV. The chemical structures of the compounds were identified by comparing the spectrum with the NIST Mass Spectral Library.

### Molecular dynamic simulation of DMADP

The DMADP structure was simulated at constant normal pressure and temperature (NPT ensemble). The models of DMADP and Mn^2+^ or Mg^2+^in the water was fabricated using “tleap” program of Amber16 (AMBER 2016, Case et al. 2016^21^, https://ambermd.org/index.php) with a force field of ions 234lm_1264_tip3p for metal ions and with gaff2 for DMADP. One DMDP molecule and three metal salts were placed in 15 Å tip3p solvate box (43.9 × 42.1 × 42.9 Å). The system was energy-minimized by 3500 steps and solvent-relaxed for 40 ps under constant volume (ntb = 1), followed by all-relaxation under constant pressure (ntb = 2, NPT ensemble) for 100 ps. The system was further equilibrated by NPT ensemble at 313 K for 100 ps, and the simulation was continued for another 10 ns. The simulation parameters were as follows: nstlim = 10,000,000, dt = 0.001, imin = 0, irest = 1, ntx = 5, ntb = 2, pres0 = 1.0, ntp = 1, taup = 2.0, cut = 12, ntr = 0, ntc = 2, ntf = 2, tempi = 313.0, temp0 = 313.0, ntt = 1, tautp = 1.0, ntpr = 100, ntwx = 100, ntwr = 100, iwrap = 1. Simulation of 10 ns (100,000 trajectories) was used for analysis. The atom distance and dihedral angle were measured using the “CPPTRAJ” program of Amber Tools.

## Results

**Figure 1:**
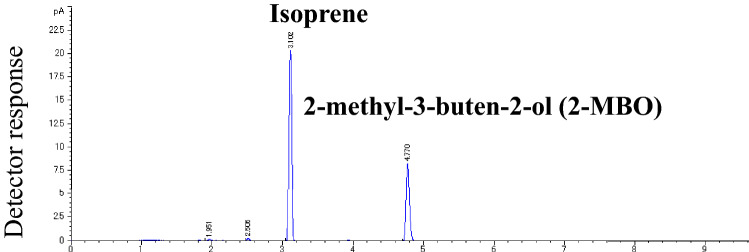
GC chromatogram of head space gas of the reaction mixture. Four mM DMADP was reacted with 20 mM Mn^2+^ at 55 °C with pH 8.5 for 1 h and the head space gas was analyzed by gas-chromatography. The first and second peaks were respectively identified as isoprene and 2-methyl-4-buten-2-ol (2-MBO) by comparing the retention time with authentic standard and the MS spectrum with NIST MS library.

Figure 1 presents the gas chromatogram of the headspace gas obtained from the reaction of DMADP with Mn^2+^. DMADP (4 mM) was reacted with Mn^2+^ (20 mM) at 55 °C for 1 h. There are two prominent peaks: one eluted at 3.10 min and another eluted at 4.77 min. The first and second peaks were respectively identified as isoprene and 2-methyl-4-buten-2-ol (2-MBO) by comparing the retention time with authentic standard and the MS spectrum with NIST MS library. (Supplementary Fig. S1). The ratio of isoprene to 2-MBO under this condition was almost 2:1. To scan the side-product of the reaction, ESI–MS analysis of the liquid phase was conducted after 90 min reaction of DMADP with Mn^2+^ at 40 °C. No accumulation of specific molecule was observed in the reaction mixture suggesting occurrence of no other side-products (data not shown).

**Figure 2:**
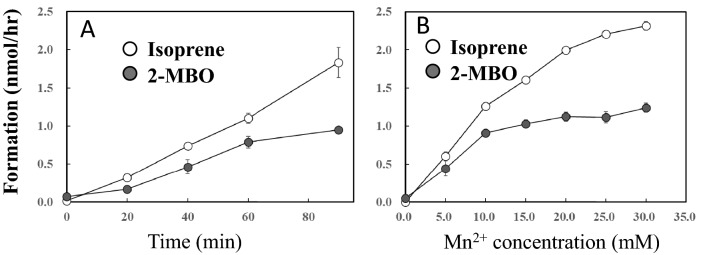
Time course (**A**) and Mn^2+^ dose dependency (**B**) of isoprene and 2-methyl-3-buten-2-ol (2MBO) formation. Data are mean ± SE (n = 3). Two mM DMADP was reacted with shown incubation time (**A**), and concentration of Mn^2+^ at 40 °C and pH 8.5 for 1 h (**B**). Head space gas was analyzed by GC. Formation of isoprene and 2MBO was calculated by relative proportion of the peak area against that of standard isoprene gas. Yield of isoprene after 60 min reaction was roughly 1% of the total amount of DMADP in the reaction mixture.

Formation of both isoprene and 2-MBO increased linearly up to 90 min incubation time (Fig. 2A). Isoprene formation increased linearly with Mn^2+^ concentration up to 20 mM and almost reached plateau level beyond this concentration whilst 2-MBO formation reached the plateau level at 10 mM (Fig. 2B). Thus, the proportion of isoprene versus 2-MBO becomes prominent at the concentration greater than 20 mM of Mn^2+^.

**Figure 3:**
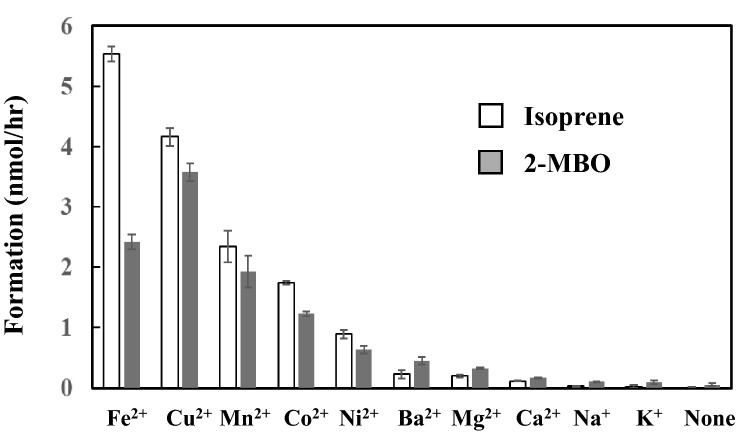
Effect of metal ions on the formation of isoprene and 2-methyl-3-buten-2-ol (2-MBO). Data are mean ± SE (n = 3). Two mM DMADP and 20 mM metal ions were reacted at 40 °C for 1 h at pH 8.5 and the head space gas was analyzed by GC. Formation rate of isoprene and 2-MBO was calculated by relative proportion against the peak area of standard isoprene gas.

Figure 3 demonstrates the effect of divalent and monovalent cations on the formation of isoprene from DMADP. The authors attempted to test the effect of trivalent cations by using AlCl_3_. However, the AlCl_3_ solution was acidic, and the pH of the reaction mixture was estimated to be less than 4.5. For this reason, its activity to generate isoprene was not investigated.

Isoprene formation was most enhanced by Fe^2+^ and, to a lesser extent, by Mn^2+^ or Cu^2+^. Ni^2+^, Co^2+^, Mg^2+^, and Ba^2+^ exhibited a relatively low activity to generate both isoprene and 2-MBO. Isoprene formation by Mn^2+^ was almost 10 times that by Mg^2+^. Monovalent cations, K^+^ and Na^+^, exhibited much less activity to generate isoprene. The proportion of isoprene *vs.* 2-MBO was higher in high-isoprene-forming metal ions, such as Fe^2+^, Cu2 + and Mn^2+^. This relationship was reversed in low-isoprene-forming metal ions, such as Mg^2+^ and Ba^2+^. Low isoprene-emitting metal ions prefer 2-MBO formation rather than isoprene formation.

**Figure 4:**
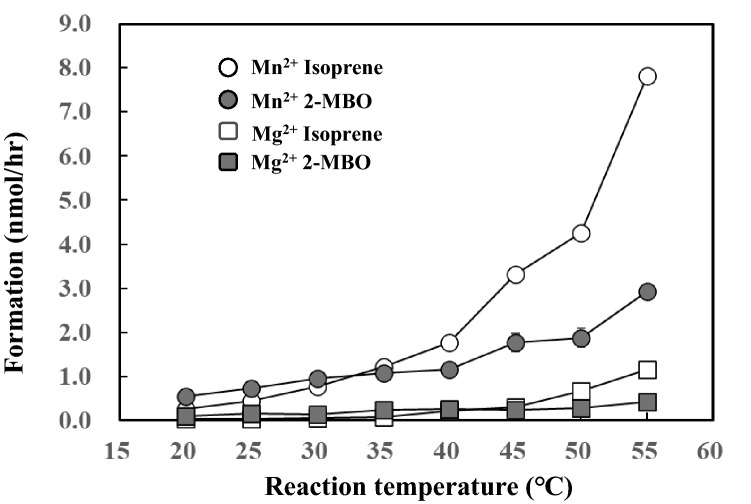
Effect of reaction temperatures on the formation of isoprene and 2-methyl-3-butene-2-ol (2-MBO). Data are mean ± SE (n = 3). Two mM DMADP and 20 mM metal ions were reacted at shown reaction temperature for 1 h with pH 8.5 and the head space gas was analyzed by GC. Formation rate of isoprene and 2-MBO was calculated by relative proportion of peak area against that of standard isoprene gas.

Isoprene formation by Mn^2+^ increased exponentially from 25 °C with temperature, as presented in Fig. 4. Furthermore, 2-MBO predominated over isoprene at a temperature less than 30 °C, whereas isoprene predominated over 2-MBO at a temperature greater than 40 °C. The Q_10_ (40 °C/30 °C) of isoprene formation was 2.3 for Mn^2+^ and 3.5 for Mg^2+^.

**Figure 5:**
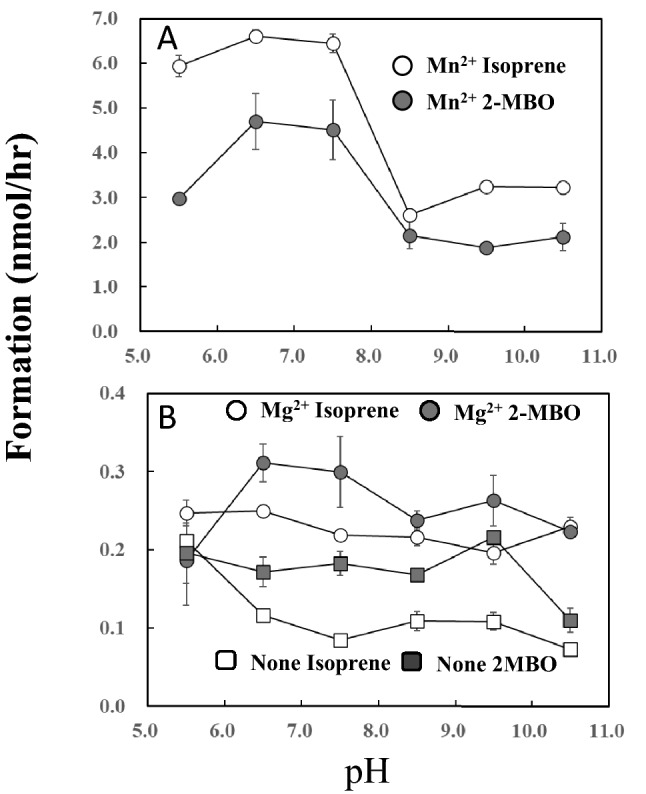
Effect of reaction pH on the formation of isoprene and 2-methyl-3-butene-2-ol (2-MBO). Data are mean ± SE (n = 3). Two mM DMADP and 20 mM metal ions were reacted at 40 ℃ for 1 h at shown pH and the head space gas was analyzed by GC. For pH 5.5 and 6.5, used buffer was 45 mM 2-(N-morpholino) ethanesulfonic acid (MES) and for pH 7.5–10.5, 45 mM Tris–HCl was used. Formation rate of isoprene and 2-MBO was calculated by relative proportion of the peak area against that of standard isoprene gas.

Formation of isoprene and 2-MBO was significantly enhanced by Mn^2+^ at acidic and neutral pH (Fig. 5), whereas no significant change was observed with Mg^2+^ or without addition of metal ions. The isoprene formation was prominent for Mn^2+^ throughout the pH variation whereas MBO was largely dominant at entire range of pH for Mg^2+^.

**Figure 6:**
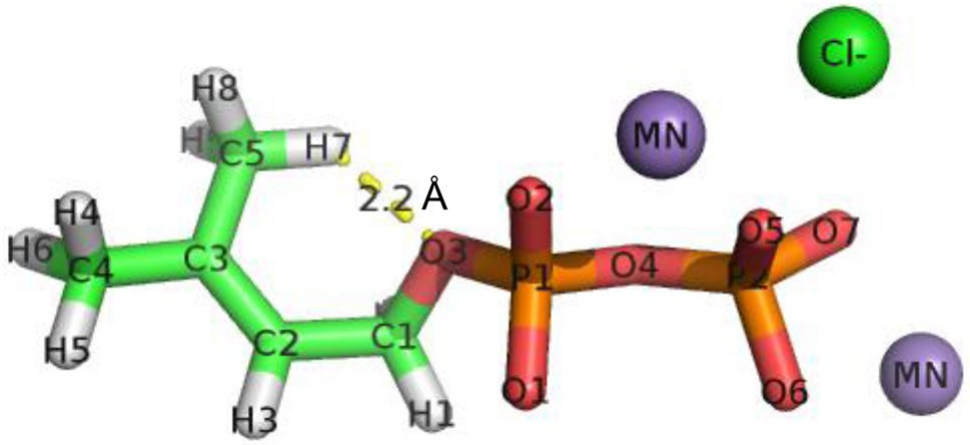
Chemical structure of DMDP bound to MgCl_2_. The DMADP structure in the water at 313 K (40 °C) was simulated for 10 ns using the Amber software. One of the models showing attractive interaction of H7 and O3 with atom distance 2.2 Å was selected. Two Mn^2+^ bound to one DMADP molecule. Three negative charges of DMADP were stoichiometrically neutralized by 2 Mn^2+^, and one excess positive charge of Mn^2+^ was antagonized by one negative chloride ion. The atom labels were by the Amber program (AMBER 2016, Case et al. 2016^21^, https://ambermd.org/index.php).

To gain more insight into the mechanism of non-enzymatic isoprene formation, the DMADP structure in the water at 313 K (40 °C) was simulated for 10 ns using the Amber software. Figure 6 presents the structure of DMADP bound with two Mn^2+^. One of the models showing attractive interaction of H7 and O3 with atom distance 2.2 Å was selected from 10 ns ensemble trajectory. Three negative charges of DMADP were stoichiometrically neutralized by two Mn^2+^, and one excess positive charge of Mn^2+^ was antagonized by one negative chloride ion (Fig. 6). The authors included three Mn^2+^ molecules in the system. One of these three Mn^2+^ was not involved in the binding to the DMADP molecule.

It has been suggested that isoprene formation could proceed via allylic carbocation, followed by quenching via base-assisted proton abstraction by analogy with the initiation step in the most related plant monoterpene synthase^22^. It has also been suggested that the oxygen (O1 or O2 in Fig. 6) of the pyrophosphate group acts as a general base, and synchronous or asynchronous allylic carbocation and deprotonation could occur in a substrate-assisted manner to form isoprene^20^. In this mechanism, phosphate oxygen (O1, O2, or O3) acts as a base to abstract the hydrogen (H4, H5, H6, H7, H8, and H9) on allyl terminal carbon (C4 or C5, referred to as ally carbon in this paper). To explore the possibility of interactions between these atoms, the NPT ensemble of the DMADP simulation comprising of 100,000 trajectories was analyzed using the CPPTRAJ program of Amber16. The authors measured the frequencies of the interaction between phosphate oxygen and allyl carbon–hydrogen with an atom distance less than 3.5 Å (Table 1). The value of 3.5 Å is the arbitral cut-off in this study and is considered to be the longest atom distance to provide attractive interatomic potential. The highest attractive interaction frequency occurred between O3 and allyl carbon–hydrogen but not between O1 or O2 and allyl carbon–hydrogen throughout all ensembles. The attractive interaction frequencies within 3.5 Å between O3 and allyl carbon–hydrogen were17 times those of the interaction between O1 or O2 and allyl carbon–hydrogen. The addition of neither Mn^2+^ nor Mg^2+^ did not induce a significant change in the interaction profile.

**Table 1:** Frequencies of interaction between phosphate oxygen and allyl carbon hydrogen with atom distance less than 3.5 Å.

Divalent cations	O3 vs allyl carbon hydrogens						O1 vs allyl carbon hydrogens						O2 vs allyl carbon hydrogens					
	H4	H5	H6	H7	H8	H9	H4	H5	H6	H7	H8	H9	H4	H5	H6	H7	H8	H9
None	0	0	0	17,627	15,789	17,280	0	0	0	803	674	948	0	0	1	1140	1138	1291
Mn^2+^	0	0	0	16,648	16,509	16,745	0	0	0	906	812	902	0	1	0	988	1003	946
Mg^2+^	0	0	0	16,864	18,900	18,415	0	0	0	1000	1152	1233	1	0	0	970	1050	1129
Fe^2+^	0	0	0	17,062	16,285	16,258	0	0	0	928	966	1098	0	0	0	1078	912	1083
Cu^2+^	0	0	0	17,101	16,417	16,836	6	1	1	1146	1111	1299	0	1	2	607	761	690

NPT ensemble for 10 ns comprised of 100,000 trajectories were analyzed by CPPTRAJ program.

The atom labels were via AMBER program (AMBER 2016, Case et al. 2016, https://ambermd.org/index.php) as shown in Fig. 6.

The average, minimum, and maximum atom distances between phosphate oxygen and allyl carbon–hydrogen has given as Supplementary Table S1. The average interaction distance between O3 and allyl carbon–hydrogen was 1.7 Å shorter than that between O1 or O2 and allyl carbon–hydrogen throughout the ensembles of control (None), Mn^2+^, Mg^2+^, Fe^2+^ and Cu^2+^. As was the case of the interaction frequencies, the atom distance profile did not exhibit any considerable difference between the control (none), Mn^2+^, and Mg^2+^ ensembles.

## Discussion

This study demonstrated the non-enzymatic formation of isoprene by Mn^2+^ for the first time. It has been proposed that allylic carbocation and quenching by deprotonation proceed in a self-catalyzed manner with pyrophosphate oxygen as the general base^20^. In this proposal, the negative charge of phosphate oxygen (O1 or O2 in Fig. 6) plays a significant role in attracting the hydrogen on terminal ally carbon (H7, H8, and H9 in Fig. 6). DMADP bears three negative charges under reaction condition of pH 8.5. Given that allyl carbon hydrogen is deprotonated via the electrostatic interaction through the negative charge of phosphate oxygen, isoprene should be formed without addition of metal ions. However, no significant formation of isoprene was observed without addition of metal ions throughout the pH range from 5.5 to 10.5 (Fig. 5). Metal ions stoichiometrically neutralizes the negative charge of oxygen as illustrated in Fig. 6 and may inhibit the electrostatic interaction between phosphate oxygen (O1 or O2 in Fig. 6) and allyl carbon hydrogen (H7, H8 or H9) hence reduce the formation of isoprene and 2-MBO. Nevertheless, isoprene and 2-MBO formation requires metal ions suggesting that the negative charge and electrostatic interaction is not involved in the deprotonation reaction. Furthermore, the molecular dynamic simulation of DMADP indicated much less attractive interaction frequencies between phosphate oxygen O1 or O2 and allyl carbon–hydrogen (Table 1 and Supplementary Table S1).

**Figure 7:**
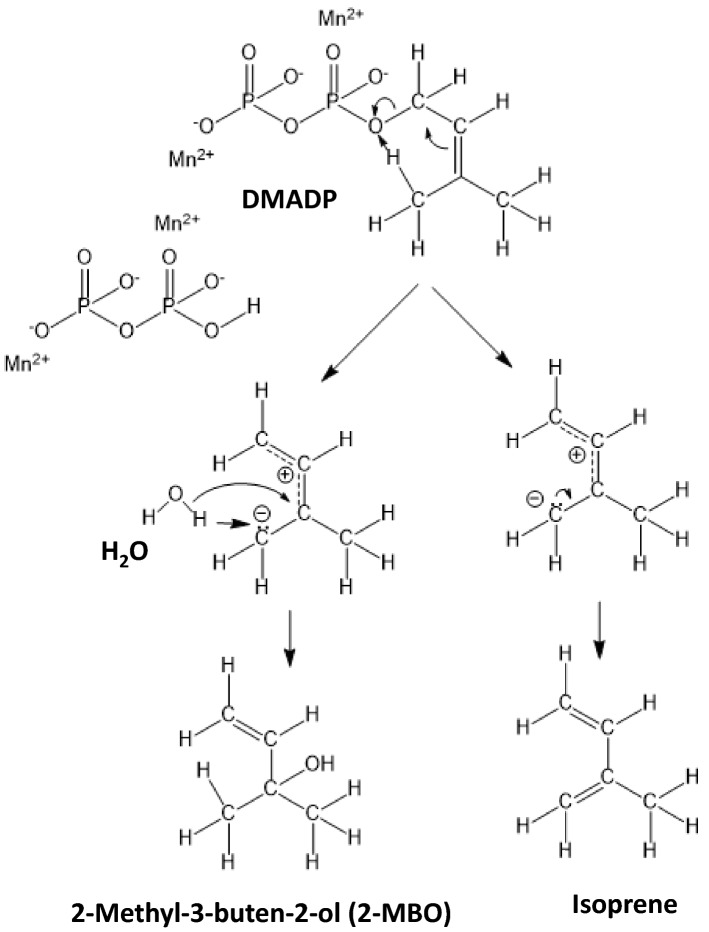
Reaction mechanism of the formation of isoprene and 2-MBO from DMADP. The formation reaction is initiated by the deprotonation of hydrogen on allyl carbon (H7, H8, and H9 in Fig. 7) by phosphate oxygen (O3). It generates carbocation and allyl anion intermediate followed by quenching to produce isoprene or by hydroxyl addition (S_N_1 reaction) to form 2-MBO.

Taking these observations together, the authors propose the isoprene and 2-MBO formation mechanism, which is illustrated in Fig. 7. The formation reaction is initiated by the deprotonation of hydrogen on allyl carbon (H7, H8, and H9 in Fig. 7) by phosphate oxygen (O3). It generates carbocation and allyl anion intermediate followed by quenching to produce isoprene or by hydroxyl addition (S_N_1 reaction) to form 2-MBO. The ratio of isoprene to 2-MBO showed variation depending on reaction conditions of time (Fig. 2A), Mn2^+^ concentration (Fig. 2B), temperature (Fig. 4) and pH (Fig. 5). Relative proportion of 2-MBO appeared to be decreased with increase in the isoprene formation in all cases. These observations therefore may be explained by trade-off of isoprene and 2-MBO formation due to competition for common precursor carbocation as illustrated in Fig. 7.

**Figure 8:**
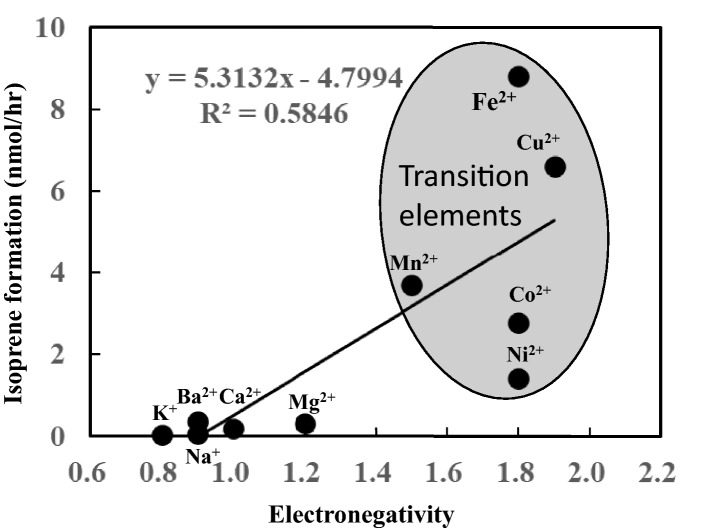
Correlation between electronegativity and isoprene formation.

Mn^2+^ exhibited high activity to induce isoprene and 2-MBO formation (Fig. 3). IspS and many other terpene synthases require Mg^2+^ or Mn^2+^metal ions as a cofactor^12,14–17^. The formation of isoprene by Mn^2+^ was almost ten times that by Mg^2+^. The authors speculated that Mn^2+^ accelerates the attractive interaction frequencies by binding to pyrophosphate. However, the molecular dynamic simulation revealed no difference in the interaction frequencies between Mn^2+^ and Mg^2+^ (Table 1). The binding of Mn^2+^ could affect the electron localization or electrostatic potential of allyl terminal carbon–oxygen (O3) to subtract allyl hydrogen and facilitate the carbocation formation. Thus, the higher electronegativity of Mn^2+^ (electronegativity = 1.5) than that of Mg^2+^ (electronegativity = 1.2) may explain the difference. However, no strong linear correlation was observed between electronegativity and isoprene formation (Fig. 8). Mn^2+^, Fe^2+^, and Cu^2+^ are transition elements, and their electron configuration in the d orbital of the M electron shell may bear some relevance with the high formation rate of carbocation. This hypothesis needs further investigation.

Here, we demonstrated the non-enzymatic formation of isoprene and 2-MBO by Mn^2+^ for the first time. Isoprene has been considered to be synthesized by only IspS in the plant kingdom. However, this study highlighted some parts of isoprene emission from terrestrial plants, which could be formed by non-enzymatic mechanism. It has been suggested that isoprene protects the photosynthetic system from thermal stress^23,24^. Moreover, it stabilizes the photosynthetic protein complex in the thylakoid membrane^25^. Elevated temperature disrupts the manganese cluster and releases manganese from its binding site^26,27^. Therefore, the authors speculate that liberated manganese in chloroplast on high temperature binds to DMADP and instantly generates isoprene and may serve as an acute safety net to protect the photosystem. However, the physiological significance of non-enzymatic isoprene formation is yet to be demonstrated.

There is growing evidence that tropical trees emit trace gases, such as 2-MBO and dimethyl sulfide^28,29^. Both isoprene and 2-MBO are produced from the common precursor DMADP. It therefore could be possible that 2-MBO emitted from terrestrial plants is formed non-enzymatically from DMADP as our proposal in this study or enzymatically via 2-MBO synthase. It has been shown that 2-MBO synthase produces 2-MBO to isoprene at a ratio of 90:1^30^. This observation suggests that isoprene synthase and 2-MBO shares the same formation mechanism. Given that the 2-MBO synthesis is also by substrate assisted mechanism as our proposal, the active site of 2-MBO synthase may provide a niche to allow entry of water. By analogy, the active site of isoprene synthase excludes water molecule to specifically enhance isoprene formation from carbocation as illustrated in Fig. 7. The hydrophilicity or hydrophobicity of active pocket could be crucial for product specificity of isoprene or 2-MBO biosynthesis.

Some of isoprene synthase from tropical tree preferred Mn^2+^ as cofactor rather than Mg^2+^^31^. Our previous^19^ and ongoing work found that 7 of 9 IspSs from tropical trees showed higher dependency on Mn^2+^ compared to Mg^2+^ (unpublished observation). The isoprene synthase kinetics and enzyme activation are an important controlling factor of isoprene emission. The dependency of IspS on Mn^2+^ therefore may relate with the isoprene emission behavior of tropical trees and this may need further investigation.

## Supplementary Information

Supplementary Information.
