# Supplementary material for: Non-enzymatic formation of isoprene and 2-methyl-3-buten-2-ol (2-MBO) by manganese
Source: Sci Rep. 2022 Feb 14;12:2465. doi: 10.1038/s41598-022-06520-0 (PMC8844067; doi:10.1038/s41598-022-06520-0)

## Slide 1
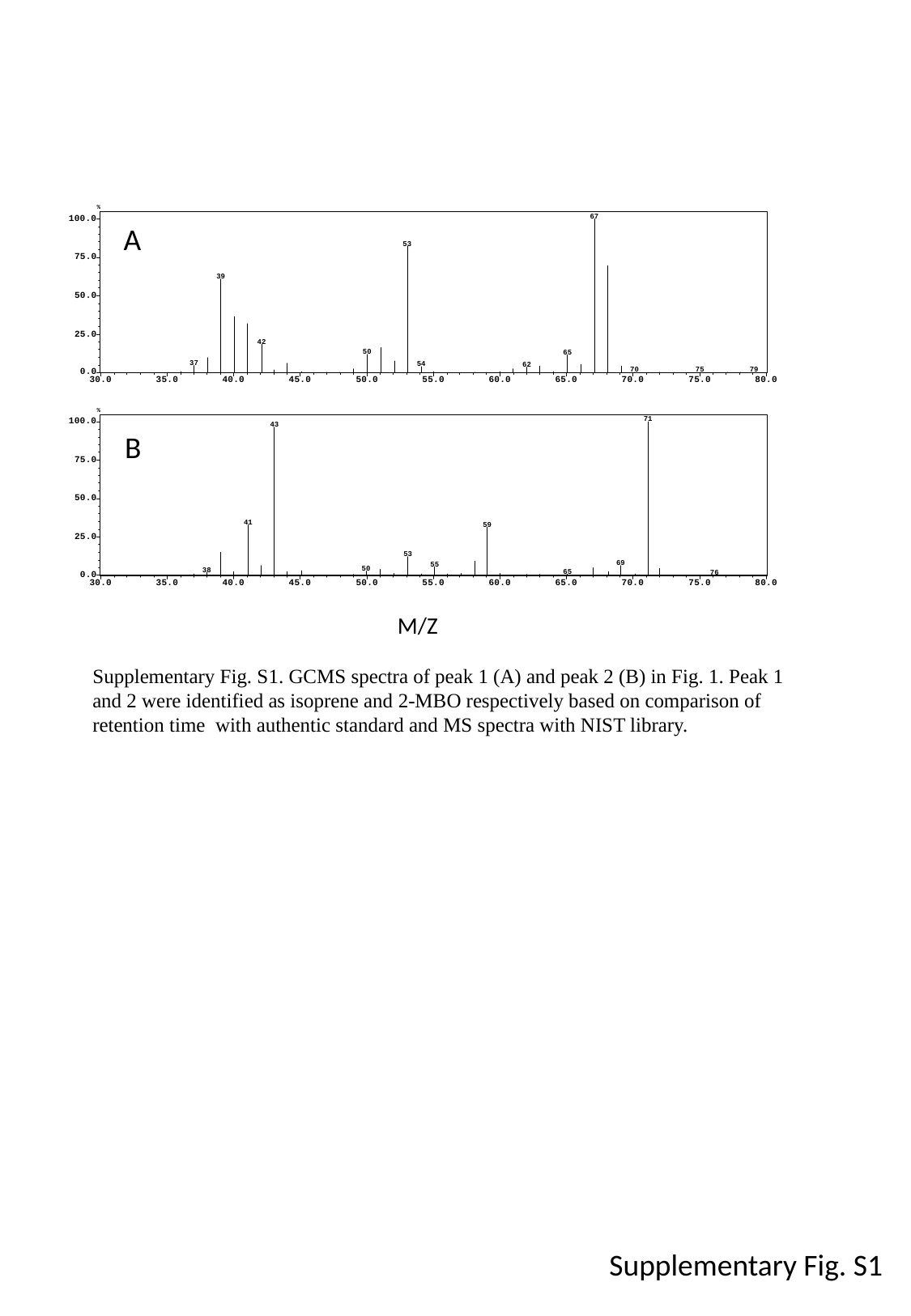

#
A
B
M/Z
Supplementary Fig. S1. GCMS spectra of peak 1 (A) and peak 2 (B) in Fig. 1. Peak 1 and 2 were identified as isoprene and 2-MBO respectively based on comparison of retention time with authentic standard and MS spectra with NIST library.
Supplementary Fig. S1

## Slide 2
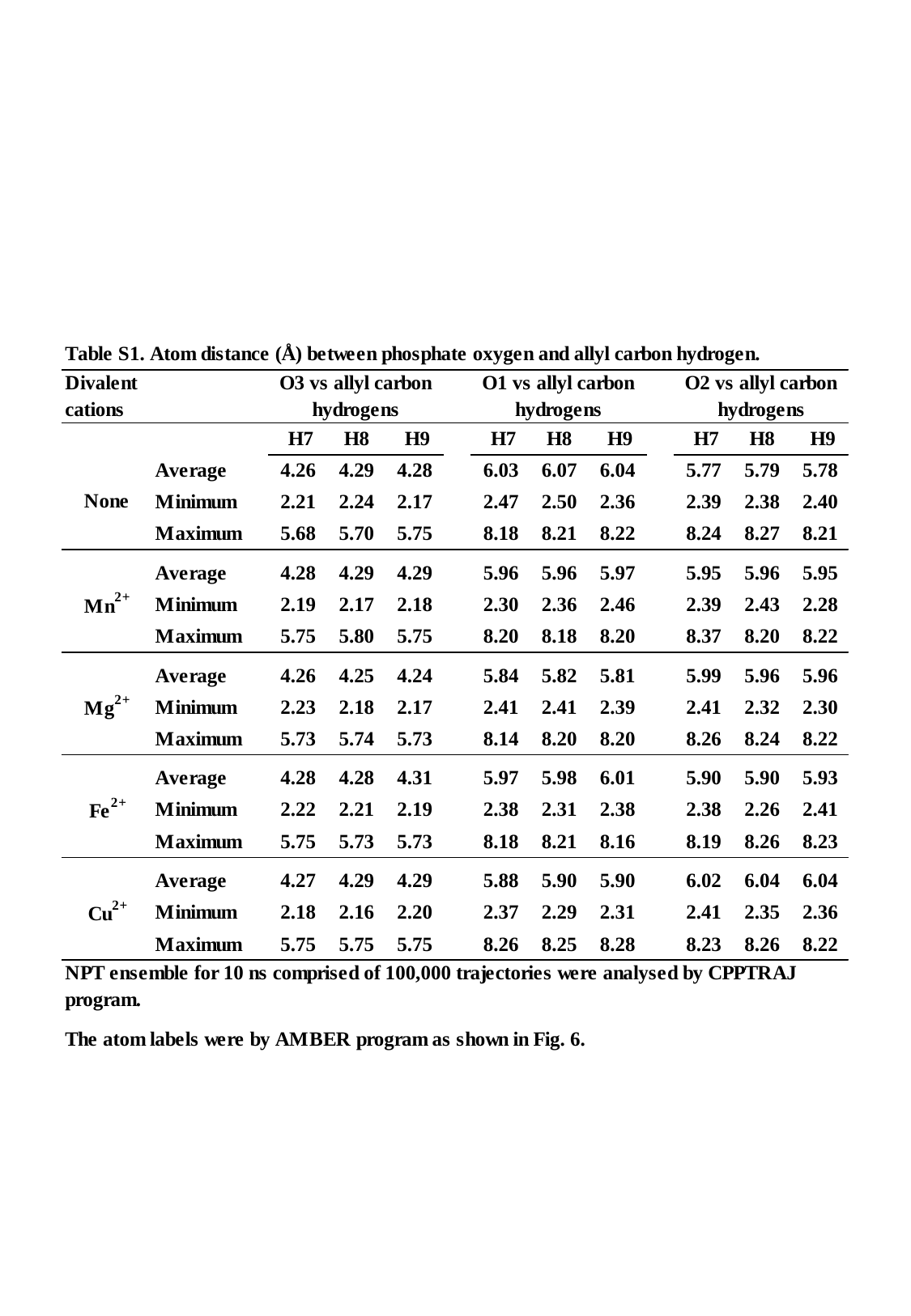

Supplement: Supplementary file 1 — Supplementary Information. [file 41598_2022_6520_MOESM1_ESM.pptx]
